# The Other Side of the Coin: Nocebo Effects and Psychotherapy

**DOI:** 10.3389/fpsyt.2019.00555

**Published:** 2019-08-08

**Authors:** Cosima Locher, Helen Koechlin, Jens Gaab, Heike Gerger

**Affiliations:** ^1^Division of Clinical Psychology and Psychotherapy, Faculty of Psychology, University of Basel, Basel, Switzerland; ^2^School of Psychology, University of Plymounth, Plymouth, United Kingdom; ^3^Department of Anesthesiology, Critical Care, and Pain Medicine, Boston Children’s Hospital, Harvard Medical School, Boston, MA, United States

**Keywords:** nocebo effects, adverse (side) effects, psychotherapy, trauma debriefing, chronic primary pain, (negative) treatment expectations

## Abstract

Psychotherapy and placebo have a long history, and both have been shown to have significant and clinically meaningful effects. In the last 100 years and up to today, psychotherapy has been subject to an enduring and often heated debate about its mechanisms and its possible relationship to placebos and their effects. However, there is little awareness of the placebo effects’ counterpart—nocebo effects (from Latin “I will harm”)—in the context of psychotherapy. Embedded in the controversy of whether psychotherapy and placebo share some unwanted proximity in terms of effects and mechanisms, the question arises which role nocebo effects may play in relation to psychotherapy. By using two examples, this article analyzes and discusses two different kinds of possible associations between psychotherapy and nocebo effects. We close with possibilities of how to prevent the occurrence of nocebo effects in psychotherapy, including some specific recommendations for clinical practice.

## Psychotherapy, Placebo, and Nocebo

Throughout its history, psychotherapy has been associated with placebos and their effects, and much of psychotherapy’s progress and controversy are owed to this complex and disputed relationship (1, 2). The debate encompasses the first origins of psychotherapy itself (3), the early and seminal publications of Rosenzweig’s so-called Dodo bird verdict of implicit common factors underlying the effects of diverse psychotherapy approaches (4), Eysenck’s provocative claims of psychotherapy not showing greater effectiveness than spontaneous remission (5) or placebo treatment (6), Fish’s concept of “Placebo therapy” (7), and the epistemological conundrum of placebo insights (8). More recently, assumingly, verum psychotherapy was shown to be only slightly more effective than (pill) placebo (9) or nondirective supportive control treatments (10, 11), and observed differences between psychotherapies or psychotherapy and control treatments are strongly influenced by their structural equivalence (12–14) and the researchers’ allegiance (15). Also, placebos with a psychotherapeutic meaning have been shown to be effective and to have effects comparable to those observed in subjects undergoing established psychotherapy treatments (16). These methodological and epistemological issues prompted Cuijpers and Cristea (17) to publish a guideline on “[h]ow to prove that your therapy is effective, even when it is not (…).” Thus, the acknowledgment and understanding of the relationship between psychotherapy and placebo is just as much problematic as it is relevant for research (18, 19) and an ethically sound clinical practice (20). But how about nocebo effects?

Kennedy was the first to mention the nocebo effect some 50 years ago (21), emphasizing that the term *nocebo* uniquely refers to a subject-related response, a reaction inherent in the patient rather than in the active drug. The nocebo effects are typically understood as the malicious counterpart of the effects of the placebo. They are usually seen as the adverse consequences to inert treatments, which are associated with a negative meaning, whereas the placebo effects are understood as a beneficial consequence of an otherwise inert treatment provided with a therapeutic meaning. Adverse consequences of treatments can manifest as so-called side effects to an active or inert treatment, nonadherence, or even discontinuation of treatment or the lack or attenuation of beneficial effects of otherwise effective treatments (22). Noteworthy, not all adverse, negative, or missing treatment responses are to be attributed to nocebo effects (22). They can also occur because of the natural course of a given disease or disorder, the unsuitability or inaptness of a particular treatment for a given clinical condition, or the lack of responsiveness of a given clinical condition to the administered treatment. Furthermore, and of course, adverse outcome could also be the consequence of treatment errors, malpractice, and unethical or harmful behavior of the practitioner or therapist (23). But if the mechanisms assumed to explain the occurrence of adverse events after treatment administration were the same that are assumed to underlie nocebo effects, this would suggest that the adverse events were related to nocebo effects.

## Mechanisms of Nocebo Effects

Several mechanisms have been described as possibly underlying nocebo effects. One of these are patients’ negative expectations. Negative expectations can be induced verbally, that is, when patients are informed about the possible occurrence of side effects, or through the behavior of the treatment provider (24). For example, a rather nonempathic, distanced therapist may induce a negative treatment outcome expectation in the patient (25). In addition, a high somatic focus (26) and the presence of certain personality traits, such as anxiety and pessimism (27), have been related to the occurrence of nocebo effects. Furthermore, classical conditioning effects may play a role, as previous (negative) experiences with the assumed medical agent may contribute to the occurrence of nocebo effects (27). The significance of classical conditioning as an essential aspect of nocebo effects has been demonstrated in pain research (28). In addition, nocebo effects have important neurobiological and emotional correlates, which are associated with changes in brain activation (29), and may play a significant role in psychotherapy.

An additional aspect that is highly relevant for clinical practice and closely linked to the generation of (negative) expectations is the so-called narrative. In each treatment setting, different narratives play a crucial role. First, patients have their own background, experiences, and belief systems that influence their narrative of both why the symptoms are present and how they should be treated (i.e., so-called client narratives or subjective illness narratives) (30). Second, treatment providers also have their expectations and a theoretical background that shape their illness narratives. Finally, depending on the treatment and next to the theory behind it, there might be manualized methods and strategies to be used in treatment (i.e., also called the healing narrative) (31). All of these narratives influence the verbal and the nonverbal communication between patients and treatment providers (32). Importantly, the narratives of patient and provider do not necessarily match. Placebo research has shown that to harness the underlying processes, an open and honest conversation about the mechanisms that underlie the respective treatment effects is key (33). However, unintentional negative suggestions, such as trivialization (e.g., “You don’t need to worry”), or focusing attention (e.g., “Are you in pain today?”) may trigger a nocebo response (32). Assumingly, patients are especially sensitive to negative suggestions, particularly in vulnerable contexts.

## Nocebo Effects in Psychotherapy

The question arises which role nocebo effects may play in the context of psychotherapy. Interestingly and relevant to our arguments, the possible negative effects of psychotherapy were common lore in the 1960s as Barlow (34) points out, “Being awakened to the possibility that one could inflict dire harm on patients during each visit to the consulting room (or even on the way to it) was an ever-present source of anxiety during those early years for many of us” (p. 13). This “dire harm” could consist of the “Pavlovian construct of transmarginal inhibition or a state of complete shutdown of the organism,” being inflicted through “intense experiences” (p. 13). Accordingly, although psychotherapy of course can have negative consequences, such as negative side effects but also nonimprovement of symptoms or even symptom worsening (34, 23), these are regrettably underreported and underinvestigated in psychotherapy research (35). Recently, however, symptom deterioration in waiting-list control groups has been described as possibly being caused by the same mechanisms that cause nocebo effects (36): The authors argue that negative expectations regarding the hypothesized inactive control treatment and the assumption that patients give up their coping strategies while waiting for a promised effective treatment have been described to explain the observed symptom deterioration. Following a similar line of argumentation, we discuss two examples to illustrate two possible associations between psychotherapy and nocebo effects, and we analyze whether symptom deterioration or nonimprovement observed in psychotherapy may be related to nocebo mechanisms. We close our article with possible recommendations on how to prevent the occurrence of nocebo effects in psychotherapy.

## The Role of Nocebo Effects in the Treatment of Chronic Primary Pain

Patients with chronic pain often suffer from symptoms that have no clear etiology (37). The population of chronic pain patients is very heterogeneous; however, they usually share the experience of a long and unsuccessful treatment history. Patients and providers strive to find a clear symptomatic cause for the pain, but although most interventions can help patients to deal with their pain, measurable pain reduction after an intervention is usually small in the long turn (38). Chronic pain is multicausal, but treatment approaches often fail to take this into account as domain-specific approaches dominate the field (39). Furthermore, patients usually see multiple physicians and specialists during their treatment odyssey, and because the etiology for most chronic pain conditions is unknown and most likely multicausal, a plausible and satisfying narrative is hard to find. This patient group also often present with a high somatic focus, a tendency to notice and report physical symptoms, which not only leads to reports of increased pain severity and disability as well as negative emotions but also likely influences provider’s negative perception of these patients (40). Also, the nosology and terminology of the condition itself are a challenge. Chronic pain conditions without a clear etiology have been labeled as functional pain, medically unexplained pain, somatoform disorder, or psychosomatic symptoms (41). However, hearing that “It’s all in your head” (as implied by the term “psychosomatic,” for example) might lead to reduced compliance and hence symptom worsening. As past research has shown that compliance to medical advice is closely linked to patients’ understanding of their illness, a new diagnostic term has far more implications than just semantics (42). The upcoming ICD-11 introduces a new diagnostic category called chronic primary pain (CPP), which emphasizes pain itself as the disease (41). This new term holds the potential to change the common understanding of chronic pain conditions and help explain why an interdisciplinary treatment approach is crucial. In experimental pain research, the occurrence of nocebo effects has been demonstrated using placebos accompanied by negative verbal suggestions (43). All of these points may contribute to induce nocebo effects, as negative expectations caused by demoralizing treatment experiences are likely to occur. In addition, the negative appraisal of pain symptoms (e.g., the assumption that pain is a threat or linked with tissue damage) and catastrophizing or rumination around pain may further contribute to the occurrence of nocebo effects. Thus, the chronic pain population is a specifically vulnerable to the occurrence of nocebo effects even without an active or inert treatment being administered.

But what do the outlined high potential for the occurrence of nocebo effects in people with chronic pain and the possibility to induce nocebo effects by simple verbal suggestion imply for the actual treatment of chronic pain patients? In this vulnerable population, a careful focus on expectations, a focus on positive effects of the treatment, and a trustful patient-provider relationship are crucial, keeping in mind a fine-grained and sensitive understanding of the several layers these conditions present with. To avoid nocebo effects in treatment, clinicians should be especially aware of past adverse experiences that their patients might have made in previous treatments (44). Additionally, studies have identified other risk factors for the nocebo effect, such as verbal suggestions of arousal and symptoms, social observation, and baseline symptom expectations (45). Considering that, in many cases, both the patient and the provider have a negatively connoted narrative about chronic pain, an open and transparent communication about their respective understanding of the development, maintenance, and handling of chronic pain appears central, ensuring an individualized treatment plan, which is crucial for the development of a shared understanding and for the creation of a more hopeful narrative of the condition itself (46). One good example is the use of metaphors to explain that pain by itself is a necessary and adaptive bodily function; however, if the system remains in a constant state of alarm, it becomes maladaptive (42, 47). As a second example, in the context of medically unexplained symptoms, it has been shown that psychotherapeutic treatments were most effective when delivered by psychotherapists (48). This finding might be because of psychotherapists focusing on patients’ individual expectations, motivations, and perceptions, which may in turn correct patients’ inaccurate understandings of their symptoms. The idea that an inaccurate understanding of chronic pain may increase chronic pain begs the question how can we best correct that inaccurate piece of knowledge? Psychology, hand in hand with other disciplines, such as biology and neurology, can contribute to a more elaborate shared narrative between patient and treatment provider and in turn may lead to the reduction of negative expectations.

In contrast, we will give an example of a psychotherapeutic treatment that has been shown to have limited benefits, and we will discuss whether the observed effects can be related to the occurrence of nocebo effects.

## The Case of Debriefing for Trauma Survivors

In 1983, Mitchell introduced Critical Incident Stress Debriefing (CISD) (49) as a crisis intervention for use with small homogeneous groups of paramedics, fire fighters, and law enforcement officers who were distressed by an exposure to some particularly gruesome event” (p. 2) (50). Initially, CISD was not thought to be a stand-alone treatment, but it soon gained popularity, was applied in different trauma populations (51), and was adopted for use in individual settings (52). Despite numerous adoptions (53–55), the main elements remained the same, that is, the trauma experience will be discussed with a focus on distinguishing between facts, cognitions, and emotions. Through the intervention, trauma survivors shall learn to judge negative reactions after trauma experience as “normal” reactions (52).

However, despite the initial enthusiasm toward trauma debriefing, several systematic reviews and meta-analyses found no evidence for the superiority of trauma debriefing over control treatments in preventing the occurrence of posttraumatic stress disorder (PTSD) symptoms in the aftermath of trauma experience (52, 56–60). On a closer look, the reviews included a number of studies that reported even an increase in PTSD symptoms after trauma debriefing compared with control treatments (61–63). Mitchell (50) argued that the negative effects of trauma debriefing in several studies can be explained by the debriefing not being implemented as manualized, that is, not within a homogeneous group setting, not with the designated trauma populations, and not with emergency staff but with trauma victims. In contrast, other researchers argued that the negative effects indicate the real danger of debriefing interventions to contribute to symptom deterioration. In this sense, it has been proposed that the negative effects may be caused by a strong pathologizing of the trauma (27), limited time for the trauma processing (64), and the creation of an expectation toward the occurrence of PTSD symptoms (59, 60).

Of the three outlined possible explanations for the failure of trauma debriefing in preventing PTSD symptoms, two can be closely related to nocebo effects. First, the information regarding potentially occurring negative reactions after trauma experience may increase the expectation of the occurrence of negative reactions, which may in turn induce the development of such negative reactions. Second, the focus on observed symptoms after trauma experience might lead to a reevaluation of the observed symptoms in the sense that the severity of the symptoms might be exaggerated, resulting in more negative evaluations of their own symptoms. In particular, persons with a stronger tendency for somatic symptoms might even be prompted toward negative reactions of their body, including emotional states, and in turn perceive and report an increase in negative reactions. The mechanisms would thus be the same as in the case of the administration of placebo pills, which lead to the experience of side effects after debriefing patients about potentially occurring side effects. Rose and colleagues have argued in this line in explaining the disappointing results of trauma debriefing in preventing PTSD symptoms in their meta-analysis (59).

Thus, the previous analysis has demonstrated that at least some of the mechanisms that have been postulated to explain the occurrence of negative outcomes after trauma debriefing are the same as those that are used to explain the occurrence of nocebo effects, suggesting a(n) (unwanted) proximity between nocebo effects and psychotherapy.

## Conclusions

Based on the discussion of whether psychotherapy and placebos share some unwanted proximity, we set out to examine possible associations between nocebo effects and psychotherapy in the present article.

First, we examined the potential for nocebo effects in patients with chronic primary pain. In this context, we identified relevant nocebo mechanisms that may occur during treatment of chronic pain, including mainly the creation of negative expectations. Thus, we conclude that patients with chronic pain may reflect a population with a particularly high risk for the occurrence of nocebo effects. However, the same arguments may hold true for other patient populations with symptoms that lack a clear etiology (e.g., medically unexplained symptoms or mental disorders, such as depression). We highlight the need for a flexible treatment approach, to address patients with preexisting treatment experiences, their negative expectations and motivations, and their subjective illness and healing narratives. Negative treatment expectations have been demonstrated to be related to negative treatment effects in other domains of health care [e.g., Ref. (65)]. The highly individualized approaches of most psychotherapeutic treatments offer the possibility to address the outlined issues. Thus, psychotherapy may be seen as a means to reduce nocebo effects in the treatment of chronic pain.

Second, we examined whether the observed occurrence of unwanted outcomes after the administration of trauma debriefing may be related to nocebo mechanisms. We conclude that at least some of the mechanisms that are assumed to be the cause of nonimprovement or even deterioration of symptoms after debriefing of trauma survivors are the same that underlie nocebo effects—most importantly, the creation of expectations regarding the occurrence of PTSD symptoms. Accordingly, just as it has been discussed in the context of other health care settings (22, 66, 67), debriefing of patients regarding possibly occurring symptoms may contribute to nocebo effects in the context of psychotherapy as well.

In terms of recommendations for clinical practice, the most relevant question is, “How can the occurrence of nocebo effects best be avoided within an ethical framework?” In the context of psychotherapeutic treatments, this essentially involves the following principles: first, to speak openly and honestly about the possible occurrence of nocebo effects in the course of psychotherapy; second, to address possible adverse responses to psychotherapeutic treatment; and third, with respect to the importance of the narrative, the choice of words should be carefully considered in treatment settings, taking into account the patient’s own background and understanding (i.e., the patient’s subjective illness narrative). In recent years, the impact of media presentations of health on individual patient’s treatment expectations gained increasing relevance (66). Therefore, discussing and possibly correcting negative expectations, which patients gained by media consumption, in relation to the occurrence of nocebo effects, need to be considered during treatment as well.

With regard to implications for research, the main question may be “How can future studies advance our knowledge of the link between nocebo effects and psychotherapy?” One of the most important issues for psychotherapy outcome research might be that negative outcomes are measured and reported. To date, however, only a minority of psychological trials reported negative outcomes, but most psychotherapists stated that negative effects do occur within psychotherapy on a regular basis (35). Of course, unwanted effects are not necessarily linked to nocebo effects, but the reporting of negative outcomes in psychotherapy research is a prerequisite for a closer examination of the risk of the occurrence of nocebo effects.

To conclude, the issue of nocebo effects, which occur as a consequence of informing patients about the prognosis of their symptoms, including the disclosure of possibly occurring adverse reactions after treatment, is subject of an ongoing debate [e.g., Refs. (67–70)]. By outlining the possible relations between psychotherapy and nocebo effects, the present article contributes to translating this debate to the field of psychotherapy research.

## Author Contributions

CL, HK, JG, and HG wrote and reviewed the manuscript.

## Funding

CL, PhD, received funding for this project from the Swiss National Science Foundation (SNSF): P400PS_180730 (Title: Overcoming Classificatory and Methodological Hurdles to Improve Treatment of Chronic Primary Pain: A Network Meta-Analytic Approach).

## Conflict of Interest Statement

The authors declare that the research was conducted in the absence of any commercial or financial relationships that could be construed as a potential conflict of interest.

## References

[1] Gaab J Blease C Locher C Gerger H Go open: a plea for transparency in psychotherapy. Psychol Conscious: Theory, Research, and Practice (2016) 3(2):175–98. 10.1037/cns0000063

[2] Kirsch I Placebo psychotherapy: synonym or oxymoron? J Clin Psychol (2005) 61(7):791–803. 10.1002/jclp.20126 15827992

[3] Crabtree A From Mesmer to Freud: magnetic sleep and the roots of psychological healing (New). New Haven: Yale University Press (1994).

[4] Rosenzweig S Some implicit common factors in diverse methods of psychotherapy. Am J Orthopsychiatry (1936) 6(3):412–5. 10.1111/j.1939-0025.1936.tb05248.x

[5] Eysenck HJ The effects of psychotherapy: an evaluation. J Consult Psychol (1952) 16(5):319–24. 10.1037/h0063633 13000035

[6] Eysenck HJ The outcome problem in psychotherapy: what have we learned? Behav Res Ther (1994) 32(5):477–95. 10.1016/0005-7967(94)90135-X 8042959

[7] Fish JM Placebo therapy: A practical guide to social influence in psychotherapy. San Francisco: Jossey-Bass (1973).

[8] Jopling DA Placebo insight: The rationality of insight-oriented psychotherapy. J Clin Psychol (2001) 57(1):19–36. 10.1002/1097-4679(20010157:1<19::AID-JCLP4>3.0.CO;2-Z 11211284

[9] Cuijpers P Turner EH Mohr DC Hofmann SG Andersson G Berking M Comparison of psychotherapies for adult depression to pill placebo control groups: a meta-analysis. Psychol Med (2014) 44(4):685–95. 10.1017/S0033291713000457 23552610

[10] Cuijpers P Driessen E Hollon SD van Oppen P Barth J Andersson G The efficacy of non-directive supportive therapy for adult depression: A meta-analysis. Clin Psychol Rev (2012) 32(4):280–91. 10.1016/j.cpr.2012.01.003 22466509

[11] Gerger H Munder T Gemperli A Nüesch E Trelle S Jüni P Integrating fragmented evidence by network meta-analysis: relative effectiveness of psychological interventions for adults with post-traumatic stress disorder. Psychol Med (2014) 44(15):3151–64. 10.1017/S0033291714000853 25066766

[12] Baskin TW Tierney SC Minami T Wampold BE Establishing specificity in psychotherapy: a meta-analysis of structural equivalence of placebo controls. J Consult Clin Psychol (2003) 71(6):973–9. 10.1037/0022-006X.71.6.973 14622072

[13] Gerger H Munder T Barth J Specific and nonspecific psychological interventions for PTSD symptoms: a meta-analysis with problem complexity as a moderator. J Clin Psychol (2014) 70(7):601–15. 10.1002/jclp.22059 24353221

[14] Locher C Hasler S Gaab J Placebos in der Psychotherapieforschung - eine systematische Analyse am Beispiel der systematischen Desensibilisierung. Verhaltenstherapie (2016) 26(1):9–20. 10.1159/000443464

[15] Gerger H Gaab J (2016). Researcher allegiance as hidden moderator in psychotherapyoutcome research. Verhaltenstherapie. 10.1159/000443543

[16] Gaab J Kossowsky J Ehlert U Locher C Effects and components of placebos with a psychological treatment rationale – three randomized-controlled studies. Sci Rep (2019) 9(1):1421. 10.1038/s41598-018-37945-1 30723231 PMC6363794

[17] Cuijpers P Cristea IA How to prove that your therapy is effective, even when it is not: a guideline. Epidemiol Psychiatr Sci (2016) 25(5):428–35. 10.1017/S2045796015000864 PMC7137591 26411384

[18] Blease CR Psychotherapy and placebos: manifesto for conceptual clarity. Front Psychiatry (2018) 9:379. 10.3389/fpsyt.2018.00379 30177892 PMC6109685

[19] Locher C Gaab J Blease C When a placebo is not a placebo: problems and solutions to the gold standard in psychotherapy research. Front Psychol (2018) 9:2317. 10.3389/fpsyg.2018.02317 30542310 PMC6277873

[20] Trachsel M Gaab J Disclosure of incidental constituents of psychotherapy as a moral obligation for psychiatrists and psychotherapists. J Med Ethics (2016) 42(8):493–5. 10.1136/medethics-2015-102986 27169707

[21] Kennedy WP The nocebo reaction. Med World (1961) 95:203–5. 13752532

[22] Colloca L Miller FG The nocebo effect and its relevance for clinical practice. Psychosom Med (2011) 73(7):598–603. 10.1097/PSY.0b013e3182294a50 21862825 PMC3167012

[23] Lilienfeld SO Psychological treatments that cause harm. Perspect Psychol Sci (2007) 2(1):53–70. 10.1111/j.1745-6916.2007.00029.x 26151919

[24] Langewitz W Placebo-Nocebo. In: Adler R Herzog W Joraschky P, editors. Psychosomatische Medizin. Theoretische Modelle und klinische Praxis. München: Urban & Fischer (2011). p. 493–8. 10.1016/B978-3-437-21831-6.10041-3

[25] Howe LC Goyer JP Crum AJ Harnessing the placebo effect: Exploring the influence of physician characteristics on placebo response. Health Psychol (2017) 36(11):1074–82. 10.1037/hea0000499 PMC7608626 28277699

[26] Tracey I Getting the pain you expect: mechanisms of placebo, nocebo and reappraisal effects in humans. Nat Med (2010) 16(11):1277–83. 10.1038/nm.2229 20948533

[27] Rief W Hofmann SG Nestoriuc Y The power of expectation – understanding the placebo and nocebo phenomenon. Soc Personal Psychol Compass (2008) 2(4):1624–37. 10.1111/j.1751-9004.2008.00121.x

[28] Bräscher A-K Witthöft M Becker S The underestimated significance of conditioning in placebo hypoalgesia and nocebo hyperalgesia. Pain Res Manag (2018) 2018:1–8. 10.1155/2018/6841985 PMC5833150 29670678

[29] Schienle A Höfler C Übel S Wabnegger A Emotion-specific nocebo effects: an fMRI study. Brain Imaging Behav (2018) 12:180–7. 10.1007/s11682-017-9675-1 PMC5814540 28210930

[30] Kleinman A The illness narratives: Suffering, healing, and the human condition. New York, NY: Basic books (1988).

[31] Locher C Meier S Gaab J Psychotherapy: a world of meanings. Front Psychol (2019) 10:460. 10.3389/fpsyg.2019.00460 30984050 PMC6448000

[32] Häuser W Hansen E Enck P Nocebo phenomena in medicine: their relevance in everyday clinical practice. Dtsch Arztebl Int (2012) 109(26):459–65. 10.3238/arztebl.2012.0459 PMC3401955 22833756

[33] Koechlin H Kossowsky J Gaab J Locher C How to address the placebo response in the prescription of SSRIs and SNRIs in children and adolescents. Expert Opin Drug Saf (2018) 17(6):537–40. 10.1080/14740338.2018.1475558 29781324

[34] Barlow DH Negative effects from psychological treatments: a perspective. Am Psychol (2010) 65(1):13–20. 10.1037/a0015643 20063906

[35] Jonsson U Alaie I Parling T Arnberg FK Reporting of harms in randomized controlled trials of psychological interventions for mental and behavioral disorders: A review of current practice. Contemp Clin Trials (2014) 38:1–8. 10.1016/j.cct.2014.02.005 24607768

[36] Furukawa TA Noma H Caldwell DM Honyashiki M Shinohara K Imai H Waiting list may be a nocebo condition in psychotherapy trials: a contribution from network meta-analysis. Acta Psychiatr Scand (2014) 130(3):181–92. 10.1111/acps.12275 24697518

[37] Treede R-D Rief W Barke A Aziz Q Bennett MI Benoliel R A classification of chronic pain for ICD-11. Pain (2015) 156(6):1003–7. 10.1097/j.pain.0000000000000160 PMC4450869 25844555

[38] van Koulil S Effting M Kraaimaat FW van Lankveld W van Helmond T Cats H Cognitive–behavioural therapies and exercise programmes for patients with fibromyalgia: state of the art and future directions. Ann Rheum Dis (2007) 66(5):571–81. 10.1136/ard.2006.054692 PMC1954607 16916856

[39] Wessely S Nimnuan C Sharpe M Functional somatic syndromes: one or many? Lancet (1999) 354(9182):936–9. 10.1016/S0140-6736(98)08320-2 10489969

[40] O’Brien EM Atchison JW Gremillion HA Waxenberg LB Robinson ME Somatic focus/awareness: relationship to negative affect and pain in chronic pain patients. Eur J Pain (2008) 12(1):104–15. 10.1016/j.ejpain.2007.04.001 PMC2730152 17524684

[41] Schechter NL Functional pain: time for a new name. JAMA Pediatr (2014) 168(8):693–4. 10.1001/jamapediatrics.2014.530 24887181

[42] Coakley R Schechter N Commentary: Chronic pain is like … The clinical use of analogy and metaphor in the treatment of chronic pain in children. Pediatr Pain Lett (2013) 15(1):1–8.

[43] Petersen GL Finnerup NB Colloca L Amanzio M Price DD Jensen TS The magnitude of nocebo effects in pain: A meta-analysis. Pain (2014) 155(8):1426–34. 10.1016/j.pain.2014.04.016 PMC4213146 24780622

[44] Data-Franco J Berk M The nocebo effect: a clinicians guide. Aust N Z J Psychiatry (2013) 47(7):617–23. 10.1177/0004867412464717 23093053

[45] Chavarria V Vian J Pereira C Data-Franco J Fernandes BS Berk M The placebo and nocebo phenomena: their clinical management and impact on treatment outcomes. Clin Ther (2017) 39(3):477–86. 10.1016/j.clinthera.2017.01.031 28237673

[46] Kaptchuk TJ Shaw J Kerr CE Conboy LA Kelley JM Csordas TJ Maybe I made up the whole thing”: placebos and patients’ experiences in a randomized controlled trial. Cult Med Psychiatry (2009) 33(3):382–411. 10.1007/s11013-009-9141-7 19597976 PMC2716443

[47] Hyland ME Hinton C Hill C Whalley B Jones RC Davies AF Explaining unexplained pain to fibromyalgia patients: finding a narrative that is acceptable to patients and provides a rationale for evidence based interventions. Br J Pain (2016) 10(3):156–61. 10.1177/2049463716642601 PMC4994776 27583142

[48] Gerger H Hlavica M Gaab J Munder T Barth J Does it matter who provides psychological interventions for medically unexplained symptoms? A Meta-Analysis. Psychother Psychosom (2015) 48:217–26. 10.1159/000380914 26022270

[49] Mitchell JT Everly GS, (2000). Psychological debriefing: theory, practice and evidence. Cambridge: Cambridge University Press pp. 71–90.

[50] Mitchell JT (2010). www.info-trauma.org/flash/media-e/mitchellCriticalIncidentStressDebriefing.pdf.

[51] Everly GS Boyle SH Lating JM The effectiveness of psychological debriefing with vicarious trauma: a meta-analysis. Stress Med (1999) 15(4):229–33. 10.1002/(SICI)1099-1700(199910)15:4<229::AID-SMI818>3.0.CO;2-M

[52] Rose S Bisson J Brief early psychological interventions following trauma: a systematic review of the literature. J Trauma Stress (1998) 11(4):697–710. 10.1023/A:1024441315913 9870222

[53] Dyregrov A Caring for helpers in disaster situations: psychological debriefing. Disaster Manag (1989) 1(2):25–30.

[54] Rose S Psychological debriefing: history and methods. J Br Assoc Counc (1997) 1(8):48–51.

[55] Lee C Slade P Lygo V The influence of psychological debriefing on emotional adaptation in women following early miscarriage: a preliminary study. Br J Med Psychol (1996) 69(1):47–58. 10.1111/j.2044-8341.1996.tb01849.x 8829399

[56] Lewis SJ Do one-shot preventive interventions for PTSD work? A systematic research synthesis of psychological debriefings. Aggress Violent Behav (2003) 8(3):329–43. 10.1016/S1359-1789(01)00079-9

[57] Mitte K Steil R Nachtigall C Eine Meta-Analyse unter Einsatz des Random Effects-Modells zur Effektivität kurzfristiger psychologischer Interventionen nach akuter Traumatisierung. Z Klin Psychol Psychother (2005) 34(1):1–9. 10.1026/1616-3443.34.1.1

[58] Rose S Bisson J Churchill R Wessely S Psychological debriefing for preventing post traumatic stress disorder (PTSD). Cochrane Database Syst Rev (2002) (2), CD000560. 10.1002/14651858.CD000560 12076399

[59] Rose S Bisson J Wessely S A systematic review of single-session psychological interventions (‘debriefing’) following trauma. Psychother Psychosom (2003) 72(4):176–84. 10.1159/000070781 12792122

[60] Van Emmerik AAP Kamphuis JH Hulsbosch AM Emmelkamp PMG Single session debriefing after psychological trauma: a meta-analysis. Lancet (2002) 360(9335):766–71. 10.1016/S0140-6736(02)09897-5 12241834

[61] Bisson JI Jenkins PL Alexander J Bannister C Randomised controlled trial of psychological debriefing for victims of acute burn trauma. Br J Psychiatry (1997) 171(1):78–81. 10.1192/bjp.171.1.78 9328501

[62] Carlier IV Voerman AE Gersons BP The influence of occupational debriefing on post-traumatic stress symptomatology in traumatized police officers. Br J Med Psychol (2000) 73(Pt 1):87–98. 10.1348/000711200160327 10759053

[63] Hobbs M Mayou R Harrison B Worlock P A randomised controlled trial of psychological debriefing for victims of road traffic accidents. BMJ (1996) 313(7070):1438–9. 10.1136/bmj.313.7070.1438 PMC2352974 8973231

[64] Zech N Seemann M Graf BM Hansen E Nocebo-Effekte – Negativwirkungen der Aufklärung. Anästhesiol Intensivmed Notfallmed Schmerzther (2015) 50(01):64–9. 10.1055/s-0040-100081 25634378

[65] Nestoriuc Y von Blanckenburg P Schuricht F Barsky AJ Hadji P Albert US Is it best to expect the worst? Influence of patients’ side-effect expectations on endocrine treatment outcome in a 2-year prospective clinical cohort study. Ann Oncol (2016) 27(10):1909–15. 10.1093/annonc/mdw266 27551051

[66] Faasse K Petrie KJ The nocebo effect: patient expectations and medication side effects. Postgrad Med J (2013) 89(1055):540. 10.1136/postgradmedj-2012-131730 23842213

[67] Wells RE Kaptchuk TJ To tell the truth, the whole truth, may do patients harm: the problem of the nocebo effect for informed consent. Am J Bioeth (2012) 12(3):22–9. 10.1080/15265161.2011.652798 PMC3352765 22416745

[68] Cohen S Preventing nocebo effects of informed consent without paternalism. Am J Bioeth (2017) 17(6):44–6. 10.1080/15265161.2017.1314056 28537823

[69] Colloca L Tell me the truth and I will not be harmed: informed consents and nocebo effects. Am J Bioeth (2017) 17(6):46–8. 10.1080/15265161.2017.1314057 PMC5545888 28537825

[70] Schoene-Seifert B Beware of nocebo-paternalism: pitfalls of tailored nondisclosure. Am J Bioeth (2017) 17(6):56–8. 10.1080/15265161.2017.1314049 28537829

